# Expanding hepatitis C virus test uptake using self-testing among men who have sex with men in China: two parallel randomized controlled trials

**DOI:** 10.1186/s12916-023-02981-w

**Published:** 2023-07-28

**Authors:** Cheng Wang, Peizhen Zhao, Ann Marie Weideman, Wenqian Xu, Jason J. Ong, Muhammad S. Jamil, Bin Yang, Joseph D. Tucker

**Affiliations:** 1 Dermatology Hospital of Southern Medical University, Guangzhou, China; 2 Southern Medical University Institute for Global Health, Guangzhou, China; 3 Guangdong Provincial Center for Skin Diseases and STIs Control, Guangzhou, China; 4 Department of Biostatistics, University of North Carolina at Chapel Hill, Chapel Hill, USA; 5 Center for AIDS Research Biostatistics Core, University of North Carolina at Chapel Hill, Chapel Hill, USA; 6 School of Public Health, Southern Medical University, Guangzhou, China; 7 Faculty of Infectious and Tropical Diseases, London School of Hygiene and Tropical Medicine, London, UK; 8 Central Clinical School, Monash University, Melbourne, VIC Australia; 9 Global HIV, Hepatitis and STIs Programmes, World Health Organization, Geneva, Switzerland; 10 University of North Carolina Project-China, Guangzhou, China; 11 Institute for Global Health and Infectious Diseases, School of Medicine, University of North Carolina at Chapel Hill, Chapel Hill, USA

**Keywords:** Hepatitis C virus, HCV, Self-testing, Men who have sex with men, MSM

## Abstract

**Background:**

HCV self-testing (HCVST) may be an effective strategy to address low rates of HCV test uptake among men who have sex with men (MSM). We evaluated the effectiveness and cost of providing HCVST to increase HCV test uptake among MSM in China.

**Methods:**

Two parallel, unmasked, individual-level randomized controlled trials were conducted. HIV-negative MSM and MSM living with HIV were enrolled from 22 cities in China. Men in both trials were randomly assigned (1:1) into standard-of-care (SOC) or HCVST arms. The primary outcome was the proportion of participants who tested for HCV during the trial period. Intervention effects were estimated using multiply imputed data in the main analysis. Costs were measured using a micro-costing approach.

**Results:**

A total of 84 men who were HIV-negative (trial 1) and 84 men living with HIV were enrolled (trial 2). Overall, the proportion of individuals who underwent HCV testing during the trial period was higher in the HCVST arm compared to SOC in trial 1 (estimated risk difference (RD): 71.1%, 95% CI: 54.6 to 87.7%) and trial 2 (estimated RD: 62.9%, 95% CI: 45.7 to 80.1%). Over half (58.6%, 34/58) of HCV self-testers reported the self-test was their first HCV test. The cost per person tested in trial 1 was $654.52 for SOC and $49.83 for HCVST, and in trial 2 was $438.67 for SOC and $53.33 for HCVST.

**Conclusions:**

Compared to the standard of care, providing HCVST significantly increased the proportion of MSM testing for HCV in China, and was cheaper per person tested.

**Trial registration:**

Chinese Clinical Trial Registry. Registration number: ChiCTR2100048379.

**Supplementary Information:**

The online version contains supplementary material available at 10.1186/s12916-023-02981-w.

## Background

An estimated 58 million people were living with hepatitis C virus (HCV) worldwide in 2019 [1]. HCV causes approximately 700,000 deaths each year [2]. China has a considerable HCV disease burden accounting for over 7% of the global cases recorded, and the HCV prevalence rates vary geographically, with the northern and western provinces having greater burden [3, 4]. Direct-acting antivirals (DAA) have improved the cure rate of HCV infection to over 95% [5], providing unique opportunities for achieving viral hepatitis elimination targets established by the World Health Organization (WHO) [2]. There is a need for testing both high-risk and low-risk individuals [6], especially in high-burden countries like China [7, 8]. However, only 34.9% of people know that they have HCV in China [9]. HCV testing rates are low among both high-risk groups as well as low-risk groups [10, 11].

MSM in China include individuals at higher risk of HCV among men living with HIV as well as lower risk populations. Clusters of HCV infection have been reported among MSM living with HIV and the prevalence of HCV among MSM with HIV is 6.3% (95% CI: 5.3 to 7.5%), and these individuals are at greater risk for HCV infection [12]. Higher risk for HCV based on factors identified by the WHO [13]. MSM without HIV infection are likely a lower risk group with a prevalence of 1.5% (95% CI: 1.0 to 2.1%) [12]. However, a 2017 nationwide survey revealed that around 60% of Chinese MSM remain untested for HCV [14]. HCV testing services have been centralized and relied on hospital outpatient facilities in China [15]. Barriers preventing MSM from accessing HCV testing include lack of knowledge about HCV, low perceived risk of HCV infection, societal marginalization, stigma and discrimination from health care providers, testing cost, limitations of facility-based HCV testing such as inconvenience and lack of privacy, and COVID-19 restrictions [16–18]. A survey on HIV testing found that the number of MSM undergoing facility-based HIV testing reduced by 59.0% (95% CI: 58.0 to 60.0%) after the COVID-19 outbreak [19]. Innovative methods for increasing HCV testing are urgently needed.

Evidence and experiences from a large body of self-testing programmes for HIV [20, 21] and syphilis [22, 23] indicate that HCV self-testing may be an effective approach to address those barriers. A global systematic review and meta-analysis showed that HIV self-testing increased the uptake of HIV testing in MSM by 1.5% (95% CI: 1.2 to 1.8%) times compared to standard HIV testing [24]. HCV self-testing is a process whereby individuals collect their own specimen (blood or oral fluid), perform the test, and interpret the result themselves [13]. Although there are currently no tests approved for HCV self-testing by WHO prequalification or other stringent regulatory authorities, several quality-assured tests are in the pipeline. To adapt and introduce self-testing for HCV, in 2019, the Foundation for Innovative New Diagnostics (FIND), in collaboration with WHO, conducted a series of pilot studies of HCV self-testing in Egypt [25], Vietnam [26], Georgia, Kenya, and China [27], which showed a high usability, acceptability and feasibility of using HCV self-testing among MSM. The pilot study in China showed that 76.0% (95% CI: 66.4 to 84.0%) of MSM found HCV self-testing acceptable [27]. These findings led to the first global HCV self-testing guideline published by WHO in 2021, recommending HCV self-testing as an additional testing approach to increase HCV testing coverage [13]. The systematic review identified no randomized controlled trials focused on HCV self-testing and highlighted the need for further implementation research [28].

We designed two parallel randomized controlled trials (RCT) to evaluate the effectiveness and cost of HCV self-testing to increase testing among MSM in China compared to standard-of-care: one in a low-risk HCV group (HIV-negative MSM) (trial 1) and one in a high-risk HCV group (MSM living with HIV) (trial 2).

## Methods

### Study design

This is a multi-site, two-parallel, unmasked, individual-level RCTs conducted in seven Chinese provinces. Eligible participants met the following criteria: born biologically male, 18 years or older, reported anal sex with other men, not tested for HCV in the past year, reporting at least one of the following risk factors in the past year (condomless anal sex or diagnosed sexually transmitted infection (STI) or injection drug use), planning to live in China for the next month, and residing at a stable residence to receive a postal package securely. Participants were excluded if they were participating in similar clinical trials or other programmes involving HCV testing or were unwilling or unable to comply with all the study requirements. Eligible men were offered enrollment into one of two trials according to their HIV status. In trial 1, we enrolled men who had an HIV-negative result according to their laboratory test report in the past 3 months. In trial 2, we enrolled men who had an HIV-positive result confirmed by a test report. Recruitment took place from December 24, 2021, to January 30, 2022. Participants in each arm were followed for 4 weeks. The trial follow-up and data collection were completed on March 14, 2022. A pilot study was conducted to evaluate the feasibility of recruitment and to provide preliminary estimates of the effect sizes to partially inform the sample size and power calculations (Additional file 1).

We acquired permission from participants to assess their IP address before the study. We reported our findings according to the Consolidated Standards of Reporting Trials (CONSORT) guidelines (Additional file 2: p 1) and the CONSERVE 2021 statement (Additional file 2: p 5). The study protocol was approved by the institutional review board before the study initiation and no changes were made to the protocol during the study. The trial was registered with the Chinese Clinical Trial Registry, number ChiCTR2100048379.

### Procedures

**Fig. 1:**
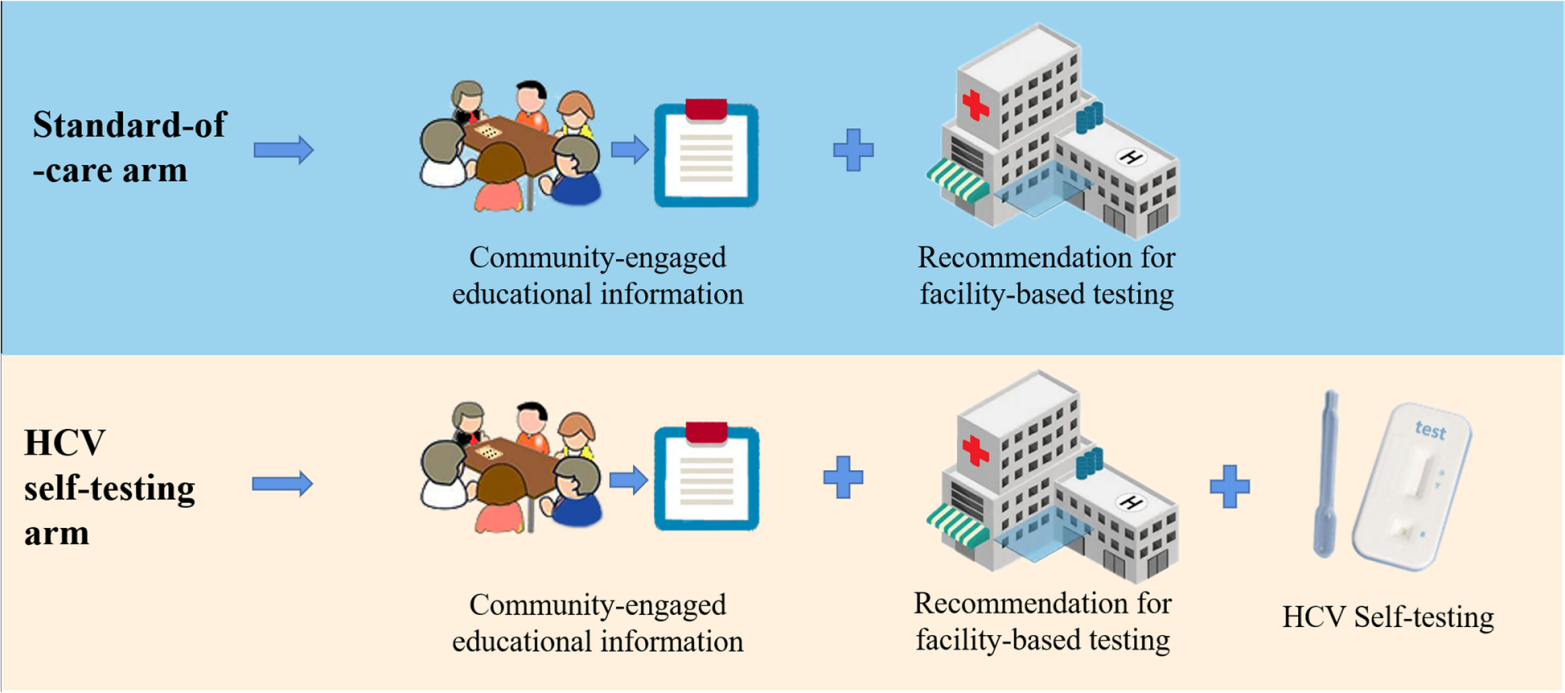
Key concepts of the interventions for each study arm

Figure 1 shows the key concepts of the interventions for each study arm.

#### Recruitment

We recruited participants by partnering with seven local MSM community-based organizations (CBOs) across seven provinces located in different parts of China. All men living with HIV were recruited in-person through MSM-led clinics, and HIV-negative men were recruited in a 50:50 ratio (half online and half offline) through CBOs. All study sites followed the same research procedure. For the offline recruitment, trained CBO staff at each clinic provided information about the study to men seeking HIV or syphilis testing services, verified the HIV status of those interested in potentially enrolling in the study, and then issued them a link to access an online survey. For the online recruitment of HIV-negative men, an invitation to join the study was posted on the WeChat Official Account of each CBO (WeChat, a popular Chinese messaging app) and was also sent via QQ (a messaging platform) by each CBO. Online recruitment was restricted to people living in the province by verifying their IP address, and all participants were required to upload their HIV-negative testing reports during the eligibility screening procedure.

All participants who clicked the link were directed to a survey website hosted by Sojump (Changsha Haoxing Information Technology Co., Ltd., China) to give consent and complete eligibility screening. Eligible participants completed a baseline survey and were required to provide contact information, including a cell phone number or a WeChat account to receive text messages, a shipping address for participants who applied for kits, and a preferred name. Participants were provided compensation of US$3 for their time completing the baseline survey.

#### Randomization and allocation

Men in both trials who completed the baseline survey and provided contact information were directed to contact a research assistant responsible for randomly allocating participants (1:1) into standard-of-care or HCV self-testing arms using a randomized block design. A separate randomization procedure was used for each CBO to generate 12 HCV self-testing/SOC randomized assignments for trial 1 and 12 assignments for trial 2. The block sizes must take the 1:1 allocation ratio into account, so for 1:1 randomization of two groups, the block sizes that can fit into 12 must be one of the even numbers 2, 4, or 6. A block size of 2 is not recommended as it is possible for the investigators to correctly guess the allocations within the small blocks and become prematurely unblinded. Thus, each of the 12 randomized assignments within each CBO was generated using two blocks of size 6 or three blocks of size 4 where the number of blocks (2 or 3) was randomly chosen based on a virtual coin flip. The final randomization schedule was generated by an independent statistician who was not involved in participant recruitment or analyses using SAS version 9.4 (SAS Institute, Cary, NC, USA).

#### Intervention

Men in the standard-of-care arm were provided with an educational message and a postcard. The message used concise language to encourage men to be tested for HCV. We invited three CBO leaders and ten community members to create the message and finalized the message based on their feedback. The postcard was developed through a national crowdsourcing contest in China in 2017 with vivid expressions to introduce the risk of acquiring HCV infection, the importance of screening for HCV, and encouragement to conduct HCV testing at local clinics [12]. The educational message and postcard were sent by SMS or WeChat at enrollment. Men in the standard of care arm could also choose to self-test for HCV, but they had to initially pay for the test, which was different from the measure in the HCV self-testing arm.

In addition to the message and postcard for men in the standard-of-care arm, men in the HCV self-testing arm were also provided with HCV self-testing services which allowed them to order a maximum of one HCV self-testing package for free through Wechat during the study period. The HCV self-testing package was sent to men by postal mail (express delivery). The package included the manufacturer-supplied step-by-step pictorial instruction in Chinese (Additional file 3: Fig. S1), an instructional video sent through Wechat and a result report card (Additional file 3: Fig. S2). Participants could upload a photo of their test results to a verification platform by scanning a barcode on the result report card which was uniquely linked to the study participant. In this study, we used a finger-prick blood HCV antibody rapid test kit (ABON, Hangzhou, China) with a 99.5% sensitivity and 99.7% specificity [29].

Study incentives were designed to be similar in the two arms. Participants in both arms were informed at enrollment that costs associated with HCV testing including facility-based HCV antibody testing and self-testing uptake during the study period could be reimbursed by providing a photo of the test report and receipt.

#### Data collection

All men in both trials were encouraged to upload their HCV testing record and results. HCV self-testing was determined by photo verification of the used test kit, while facility-based HCV testing and results were determined by photo verification of the test report. Men who uploaded photo verification were provided with US$1.

All participants completed a baseline questionnaire at enrollment, including socio-demographic characteristics, sexual behaviours, knowledge of HCV, stigma associated with HCV, attitudes towards people living with HCV, and HIV/STI testing experiences. The follow-up survey collected information on HCV testing and other STI testing experiences in the past 4 weeks. Participants were provided with a compensation of US$4 for completing the follow-up survey.

### Outcomes

The primary outcome was the proportion of participants who tested for HCV at either a clinic or via self-test during the study period. This was assessed in all participants using photo verification. Secondary outcomes included adverse outcomes related to HCV self-testing, the number of newly identified HCV infections during the trial, the linkage to HCV clinical care after self-testing, and the proportion of tests for HIV and other STIs (syphilis, gonorrhoea, and chlamydia) during the trial. The secondary outcomes were assessed based on self-reported data from a follow-up survey. We also reported the economic cost of a person tested for HCV in all study arms.

### Statistical analysis

#### General approach

Descriptive analyses were utilized to report participants’ demographic and behavioural characteristics in each study arm. Risk differences and corresponding 95% CIs were expressed as percentages instead of the standard decimal form for ease of interpretability. All inferential tests were two-sided with a type 1 error level of 0.05, and analyses were conducted using SAS version 9.4. Details on the sample size calculations and pre-specified analyses can be found in the protocol (Additional file 1).

#### Analysis using multiply imputed and complete case data

**Fig. 2:**
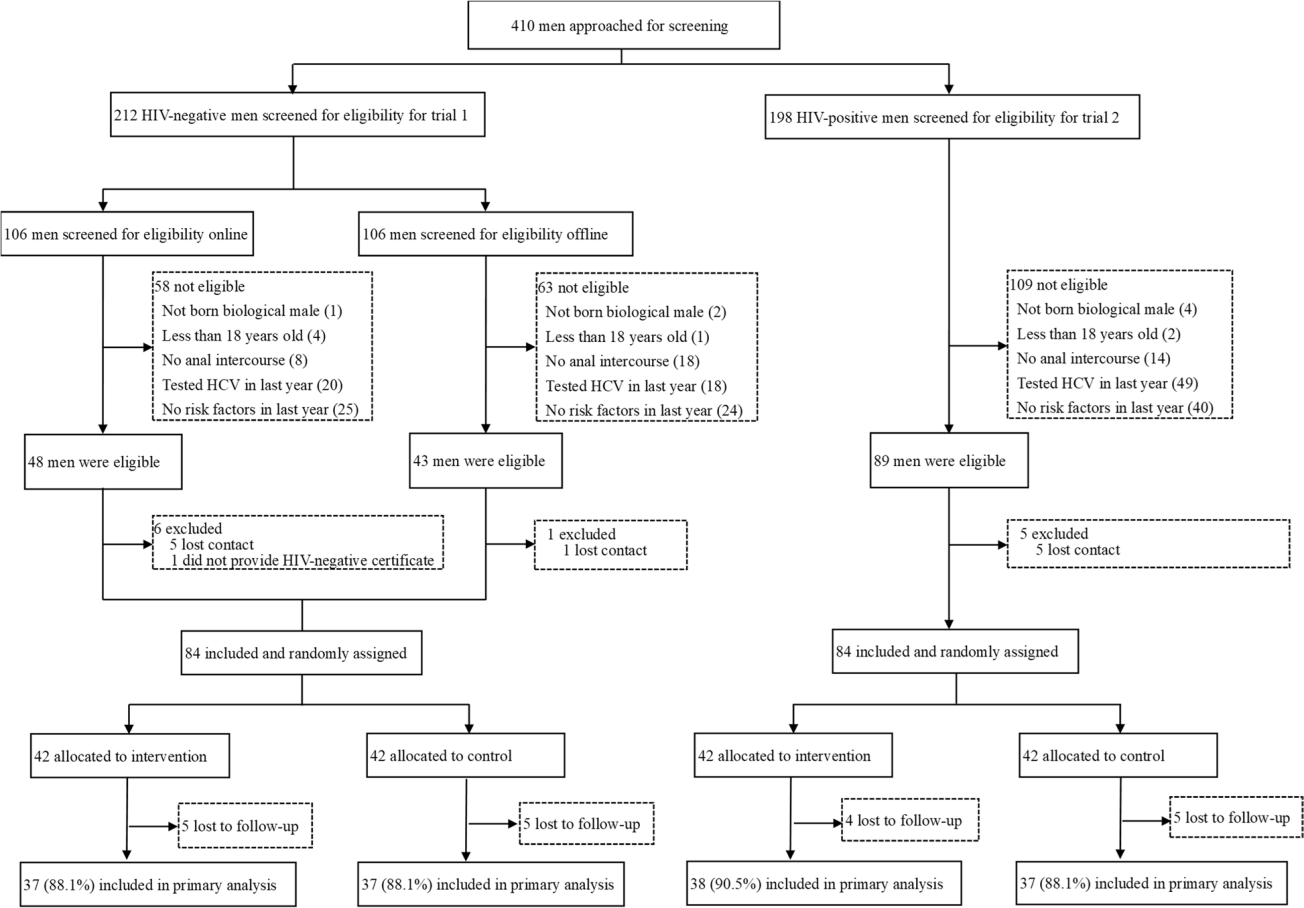
CONSORT flow chart

As pre-specified in the protocol (Additional file 1), since the primary outcome was missing for approximately 12% (over 11%) of participants in both trials (Fig. 2), analyses of primary and secondary endpoints were reported using multiple imputation to create a complete dataset for the main analysis. This was followed by a sensitivity analysis using only the complete case data (those data for which there were no missing values).

For multiple imputation, multiply imputed datasets were first generated using separate imputation models for each trial. Compared to those who completed the follow-up survey, participants who were lost to follow-up differed on several baseline demographic characteristics, also called auxiliary variables, included in each imputation model (Additional file 4: Table S1). In addition to auxiliary variables, each imputation model contained terms for the arm and interactions between arm and the auxiliary variables to account for differential loss patterns between the two arms.

We observed a monotone missing data pattern for the primary and secondary outcomes, i.e., if one of the outcomes was missing, then all other outcomes (gathered from the follow-up survey) were also missing. Multiple imputation by sequential regression was performed such that the primary outcome was imputed first given the observed data (auxiliary variables and their interaction terms with arm). Then, this imputed primary outcome was used as a predictor in the next model to impute one of the secondary outcomes; this process continued so that the final model for the last secondary outcome utilized all previously imputed outcomes as predictors.

The number of multiply imputed datasets ($\mathrm{M}$) was selected based on the maximum fraction of incomplete observations [30]. This fraction ranged from 11 to 12%, suggesting that we needed $\mathrm{M}\ge 12$, so we chose $\mathrm{M}=50$ as the number of required multiply imputed datasets. Each of the $\mathrm{M}$ datasets was analysed separately using PROC FREQ in SAS (SAS Institute, Cary, NC), and then the resulting point estimates and standard errors from each imputed dataset were combined (pooled) using Rubin’s rules [31] implemented in PROC MIANALYZE.

#### Primary outcome

The primary outcome of HCV testing uptake was evaluated using a risk difference. Point estimates and corresponding 95% Wald CIs and *p*-values were calculated and displayed in a forest plot with a vertical reference line plotted at the null hypothesis.

#### Secondary outcomes

Secondary outcomes related to testing rates for HIV and other STIs were evaluated using the same approach described for the primary outcome. Other secondary outcomes were summarized in counts and/or proportions. The magnitude of these effects was presented graphically using a forest plot.

#### Economic evaluation

We calculated each arm’s fixed and variable economic costs using a micro-costing approach. We annualized cost items (e.g. computers) using a 3% rate over the item’s lifespan. We excluded direct research-related costs. We present the total economic costs, cost per person tested, and the incremental cost-effectiveness ratios (ICER) of HCV self-testing compared with SOC for each trial. The currency exchange rate was RMB 1 = US$ 0.157, using OANDA (January 2022).

## Results

### Study participants

Overall, 410 men in the two trials were approached for screening. In trial 1, 212 HIV-negative men were approached for screening, 121 were ineligible, and an additional seven men were excluded for failing to provide contact information (*n* = 6) and HIV-negative test report (*n* = 1). A total of 84 men were finally included and randomly assigned in trial 1 (Fig. 2). In trial 2, 198 men living with HIV were approached for screening, 109 were ineligible, and an additional 5 men were excluded for failing to provide contact information. In total, 84 men were finally included and randomly assigned in trial 2. Details on the reason for exclusion can be found in Fig. 2.

### Randomization and follow-up

In both trials, 42 men were randomly assigned to the standard-of-care arm, and 42 men to the HCV self-testing arm. In trial 1, 35 (83.3%) men in the HCV self-testing arm applied for the HCV self-testing kits. Thirty-seven (88.1%) men in the HCV self-testing arm and 37 (88.1%) in the standard-of-care arm completed the follow-up survey and were included in the complete case analysis (Fig. 2). Among them, 41.9% (31/74) uploaded a photo of their test results. Characteristics of participants lost to follow-up (10 men) differed in annual income from those who completed the follow-up survey (74 men) during the trial period; individuals who completed follow-up tended to have an annual income of US$5601–9500 (47.3%, *P* < 0.05) (Additional file 4: Table S1). In trial 2, 30 (71.4%) men in the HCV self-testing arm applied for the HCV self-testing kits. Thirty-eight (90.5%) men in the HCV self-testing arm and 37 (88.1%) in the standard-of-care arm completed the follow-up survey and were included in the final analysis (Fig. 2). Among them, 40.0% (30/75) uploaded a photo of their test results. Characteristics of participants lost to follow-up (nine men) differed in marital status from those who completed the follow-up survey (75 men) during the trial period, with individuals completing follow-up tending to be never married (90.7%,* P* < 0.05) (Additional file 4: Table S1).

### Primary outcome

**Fig. 3:**
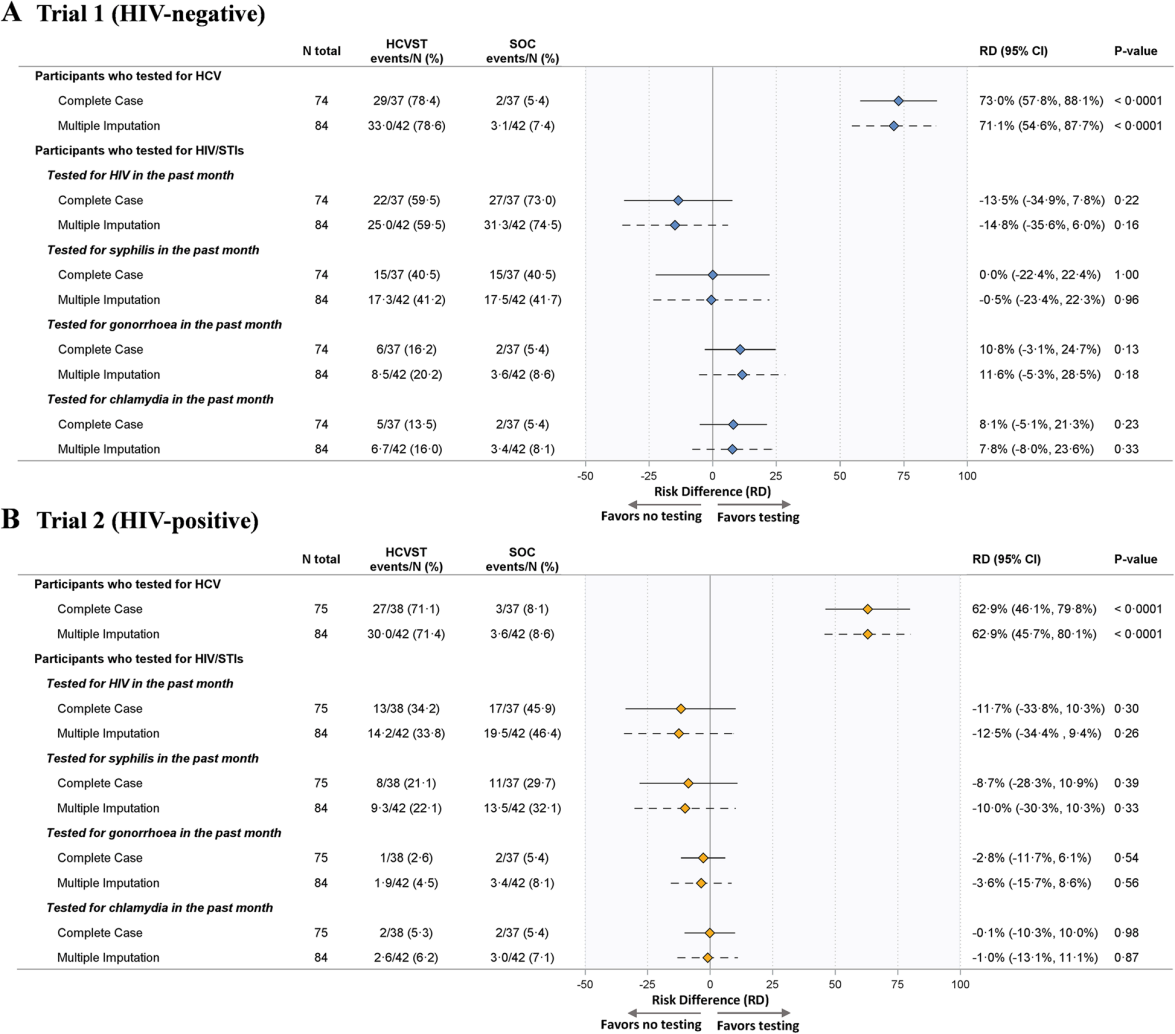
Primary and secondary outcomes for trial 1 (**A**) and trial 2 (**B**)

Key social-demographic and behavioural characteristics were similar across the two arms in both trials, except for the marital status of men living with HIV (trial 2), shown in Table 1. Overall, for the complete case analysis of trial 1, 29 of 37 men (78.4%) in the HCV self-testing arm underwent HCV testing during the trial period compared to 2 of 37 men (5.4%) in the standard-of-care arm (Fig. 3A). One man in the standard-of-care arm underwent HCV facility-based testing. When evaluated using the multiply imputated data, the proportion of individuals who underwent HCV testing during the trial period was considerably higher in the HCV self-testing arm than the standard-of-care (risk difference (RD): 71.1%, 95% CI: 54.6 to 87.7%, *p* < 0.0001). Similarly, for the complete case analysis of trial 2, 27 of 38 men (71.1%) in the HCV self-testing arm underwent HCV testing during the trial period compared to 3 of 37 men (8.1%) in the standard-of-care arm (Fig. 3B). Two men in the standard-of-care arm underwent HCV facility-based testing. Again, when evaluated by using the multiply imputed data, the proportion of individuals who underwent HCV testing during the trial period was considerably higher in the HCV self-testing arm than the standard-of-care (RD: 62.9%, 95% CI: 45.7 to 80.1%, *p* < 0.0001). In trials 1 and 2, there were no observable differences between the HCV self-testing and standard-of-care arms in HIV or STI testing rates within the trial period (Fig. 3A, B).

**Table 1:** Baseline social-demographic and behavioural characteristics of men who have sex with men in China

	**Men who were HIV-negative (trial 1)**			**Men who were HIV-positive (trial 2)**		
	**Standard-of-care (*N* = 42)**^**a**^	**Standard HCVST (*N* = 42)**^**a**^	***P*-value**^**b**^	**Standard-of-care (*N* = 42)**^**a**^	**Standard HCVST (*N* = 42)**^**a**^	***P*-value**^**b**^
**Age (years)**			0.595			0.269
≤ 30	32 (76.2)	34 (81.0)		22 (52.4)	27 (64.3)	
> 30	10 (23.8)	8 (19.0)		20 (47.6)	15 (35.7)	
Mean (SD)	27.4 (6.9)	26.6 (8.0)		32.2 (8.3)	28.8 (6.8)	
**Marital status**			0.500			0.024
Never married	36 (85.7)	38 (90.5)		33 (78.6)	40 (95.2)	
Ever married	6 (14.3)	4 (9.5)		9 (21.4)	2 (4.8)	
**Annual income (US$)**			0.072			0.840
< 2800	7 (16.7)	7 (16.7)		6 (14.3)	7 (16.7)	
2800–5600	3 (7.1)	5 (11.9)		7 (16.7)	5 (11.9)	
5601–9500	17 (40.5)	19 (45.2)		16 (38.1)	13 (31.0)	
9501–15,000	11 (26.2)	6 (14.3)		8 (19.0)	9 (21.4)	
≥ 15,001	4 (9.5)	5 (11.9)		5 (11.9)	8 (19.0)	
**Highest education**			0.825			0.172
High school or below	24 (57.1)	25 (59.5)		30 (71.4)	24 (57.1)	
College or beyond	18 (42.9)	17 (40.5)		12 (28.6)	18 (42.9)	
**Disclosure as MSM to family, friends, or healthcare professionals**			0.251			0.620
Never	12 (28.6)	17 (40.5)		10 (23.8)	12 (28.6)	
Ever	30 (71.4)	25 (59.5)		32 (76.2)	30 (71.4)	
**Number of male sex partners in the past three months**			0.510			0.653
0–1	20 (47.6)	17 (40.5)		17 (40.5)	15 (35.7)	
Multiple	22 (52.4)	25 (59.5)		25 (59.5)	27 (64.3)	
Mean (SD)	2.2 (1.8)	2.4 (2.2)		2.1 (2.0)	2.2 (1.9)	
**Anal sex without the use of condom in the past 6 months**			0.493			0.672
No	5 (16.1)	8 (22.9)		10 (31.3)	13 (36.1)	
Yes	26 (83.9)	27 (77.1)		22 (68.8)	23 (63.9)	
**Ever tested for HBV**			0.124			0.827
No	27 (64.3)	20 (47.6)		22 (52.4)	23 (54.8)	
Yes	15 (35.7)	22 (52.4)		20 (47.6)	19 (45.2)	

^a^Data are *n* (%) unless otherwise indicated

^b^*p*-values computed using a two-sided Fisher’s exact test

For the complete case data, of the 31 participants tested for HCV in trial 1, 30 men (96.8%) used HCV self-testing, and 19 of these 30 (63.3%) reported that the self-test was their first ever HCV test. One of these 31 participants (3.2%) in the HCV self-testing arm was newly identified as infected with HCV through this study and reported receiving further confirmatory testing and treatment. In trial 2, of the 30 participants tested for HCV, 28 (93.3%) men used HCV self-testing, and 15 of these 28 (53.6%) men reported that the self-test was their first ever HCV test.

### Secondary outcomes

Among the 58 men who had self-tested in the complete case analysis, one HIV-negative man reported experiencing physical violence related to HCV self-testing, which was from a regular male sexual partner. One man living with HIV reported experiencing psychological abuse related to HCV self-testing without reporting from whom (Additional file 4: Table S2).

### Cost-effectiveness

Table 2 summarizes the economic evaluation. The cost per person tested in trial 1 was $654.52 for SOC and $49.83 for HCV self-testing. The cost per person tested in trial 2 was $436.33 for SOC and $53.33 for HCV self-testing. The ICER for trial 1 was $5.02 per additional person tested, and for trial 2 was $5.46 per additional person tested using HCV self-testing compared with SOC. Further details of the cost items were included in the Appendix (Additional file 4: Table S3).

**Table 2:** Economic evaluation of standard of care compared with HCVST in men who have sex with men

	**Cost (US$)**	**Number of people tested**	**ICER**
*Trial 1 (HIV-negative men)*			
SOC	1,309	2	
HCVST	1,445	29	5.02
*Trial 2 (men living with HIV)*			
SOC	1,309	3	
HCVST	1,440	27	5.46

*HCVST* HCV self-testing arm, *ICER* Incremental cost-effectiveness ratio, *SOC* Standard-of-care arm, *US$* United States dollar

## Discussion

Our randomized controlled trial found that providing HCV self-testing among MSM substantially increased HCV test uptake compared with the standard of care. Our data suggested minimal harm associated with HCV self-testing. This study expands the literature by including people at higher risk and lower risk for HCV, leveraging digital methods for community engagement and implementation, using an RCT, and examining the cost of HCV self-testing. Findings from this study provide evidence to support the implementation and scale-up of HCV self-testing programmes among MSM.

Our results showed that HCV self-testing increases HCV testing uptake among MSM living with HIV. Numerous studies have demonstrated that MSM living with HIV have a higher HCV prevalence compared to individuals who do not have HIV [11, 32]. The greater likelihood of coinfection could be explained by high-risk practices like sexualized drug use (chemsex) and because coinfection with HIV increases the viral load of hepatitis C [32, 33]. While some high-income countries have expanded HCV testing among people living with HIV [34], most countries have low rates of HCV testing among MSM living with HIV [35]. Moreover, studies showed high rates of HCV reinfection among MSM living with HIV following HCV treatment due to ongoing risk behaviours [36, 37], suggesting the importance of repeat HCV testing among this population [11]. The 2021 WHO guidelines support the use of HCV self-testing among people living with HIV as a priority group [13]. Our RCT data provide useful implementation strategies for increasing HCV testing in this population.

Our study also showed that HCV self-testing increases HCV testing uptake among a lower risk group. This finding is consistent with several studies on self-testing for HIV [20, 21] and syphilis [22] among MSM. With DAA therapy becoming more widely available and a declining cost of treatment, public health efforts have focused on improving testing coverage and frequency in recent years [6]. The United States now recommends routine HCV testing for all adults [38]. Studies found that a routine testing strategy was highly cost-effective when coupled with DAA therapy to cure those patients identified with HCV infection [39, 40]. HCV self-testing can facilitate routine testing among lower-risk groups [25, 26].

Additionally, over half (58.6%) of HCV self-testers reported that the self-test was their first ever HCV test. This indicates that HCV self-testing has the potential to reach those who may not otherwise test. This is particularly important during the COVID-19 pandemic when facility-based testing is diminished or non-existent. Together with high acceptability and rare occurrence of social harms associated with HCV self-testing, which is consistent with data from the WHO guidelines on HCV self-testing and extensive HCV self-testing implementation research [13], this study provides strong evidence that HCV self-testing could be strategically implemented to support the achievement of the overall goal of HCV elimination. Future implementation research on linkage to clinical services is essential to ensure the full benefit of self-testing approaches. Several implementation strategies from HIV/STD (e.g., financial incentives, pay-it-forward, and peer support) may be useful to enhance linkage after HCV self-testing [41].

We found that HCV self-testing is cheaper per person tested compared to facility-based testing. The cost of testing has been identified as an important barrier to HCV testing [13]. Whilst our study did not evaluate a long enough time horizon to capture the benefits of treatment, we provide evidence to support the findings of a modelling study by the WHO which explored the cost-effectiveness of HCV self-testing in China, Georgia, Vietnam and Kenya [42]. The cost-effectiveness of HCV self-testing is driven by its potential to increase the number of people tested so that they may be successfully treated to eliminate onward transmission; this ultimately reduces the HCV burden in a community.

Our study has several limitations. First, COVID-19 regulations restricting access to facility-based testing in China might have increased demand for decentralized testing. However, during this study, the mean number of reported COVID-19 cases in the seven study sites was two people. There were no severe lockdowns during this period in the study sites and HCV services were likely near normal [43]. Second, in both arms, we reimbursed all the participants for the costs of facility-based HCV antibody testing and HCV self-testing uptake during the study period. We selected this design from a pragmatic point of view so that the data generated from the trial could help inform which testing strategy should be promoted more widely. The financial incentives to test were identical in the two arms. Third, while we used a photo-verification method to confirm the primary outcome, there exists a potential to underestimate the outcome if participants did not upload their HCV test results. However, this would bias our results towards null findings (i.e., no effect of intervention) [22]. Fourth, our study only identified one person being infected with HCV in the low-risk group (HIV-negative MSM). The lower HCV prevalence among MSM living with HIV may be related to the fact that participants already knew their negative status as part of regular HIV medical care [44]. Additionally, people infected with HCV may be more likely to be lost to follow-up and not report their positive result [11]. Future research could consider strengthening the identification of people with HCV through providing more accessible linkage-to-care services such as confirmatory testing and treatment. Fourth, building a model with a longer time horizon would enable further evaluation of the cost-effectiveness of HCV self-testing by providing data on cost per quality-adjusted life years gained or disability-adjusted life years averted.

## Conclusions

In conclusion, this RCT demonstrates the effectiveness of HCV self-testing in substantially increasing HCV testing uptake among people of higher and lower HCV risk in China. As the WHO has set the goal of eliminating viral hepatitis by 2030, scaling-up HCV self-testing among the at-risk population could play an important role in achieving this ambitious target. Future studies on optimized linkages are warranted to ensure that all men performing HCV self-testing receive timely access to HCV confirmatory testing and treatment services.

## Supplementary Information

**Additional file 1.** Study protocol.

**Additional file 2.** CONSORT Checklist and CONSERVE Checklists.

**Additional file 3: Fig. S1.** Manufacturer-supplied step-by-step instructions. **Fig. S2.** Result report card.

**Additional file 4: Table S1.** Baseline characteristics of study participants stratified by loss-to-follow-up in the HCV Self-Testing Randomized Controlled Trial in China in 2020. **Table S2.** Adverse outcome related to HCV self-testing characteristics among MSM in China (*n*=2). **Table S3.** Costs items (in 2022 USD) over a 2-month time-horizon.

## Data Availability

All deidentified data, survey instruments, and informed consent documents are available upon request.

## Abbreviations

CBOs: Community-based organizations
DAA: Direct-acting antivirals
FIND: Foundation for Innovative New Diagnostics
HCV: Hepatitis C virus
HCVST: HCV self-testing
ICER: Incremental cost-effectiveness ratios
MSM: Men who have sex with men
RCT: Randomized controlled trials
RD: Risk difference
SOC: Standard-of-care
STI: Sexually transmitted infection
WHO: World Health Organization
