# Supplementary material for: Expanding hepatitis C virus test uptake using self-testing among men who have sex with men in China: two parallel randomized controlled trials
Source: BMC Med. 2023 Jul 28;21:279. doi: 10.1186/s12916-023-02981-w (PMC10386771; doi:10.1186/s12916-023-02981-w)
Supplement: Supplementary file 1 — Additional file 1. Study protocol. [file 12916_2023_2981_MOESM1_ESM.docx]

**Additional file 1**

# Study protocol (version 1.0, completed on November, 29^th^, 2021)

**Expanding HCV test uptake using HCV self-testing among men who have sex with men in China: study protocol for two parallel randomized controlled trials**

**Abstract**

**Background:** Men who have sex with men (MSM) bear a disproportionate burden of infection with Hepatitis C virus (HCV), especially for MSM living with HIV. The low rate of HCV testing is the major public health issue among MSM in many low- and middle-income countries. We designed this study to evaluate the effectiveness and cost-effectiveness of providing HCV self-testing (HCVST) to increase HCV testing uptake among HIV-negative MSM and MSM living with HIV in China.

**Methods:** Two parallel, unmasked, individual-level, randomized controlled trials will enroll MSM who had an HIV-negative result report in the past three months (trial 1) and MSM who had an HIV-positive result report (trial 2) from seven community-based organizations (CBO) in seven provinces in China. In each trial, participants will be randomly assigned in a 1:1 ratio into one of two arms: the standard of care (SOC) arm and the HCVST arm. The SOC arm will receive a community-based message and postcard to encourage them to test for HCV at local clinics. In addition to the message and postcard for participants, we will provide free HCVST kits to participants in the HCVST arm. All MSM will complete a baseline survey and a four-week follow-up survey. HCV test results will be determined by photo verification through a digital platform.

**Conclusion:** Findings from this study will be used to explore whether HCVST can increase testing rates compared with traditional facility testing services and improve the early diagnosis rate of HCV among MSM.

**Trial registration:** Chinese Clinical Trial Registry, ChiCTR2100048379.

**Key words:** HCV, Self-testing, Randomized controlled trial, Men who have sex with men

**Background**

HCV is a public health concern with a global goal to be eliminated by 2030 set by World Health Organization (WHO)[1]. In 2019, there were 58 million people with chronic HCV infection globally, but only 21% were diagnosed[1]. Untreated HCV infection can lead to death due to liver cancer, fulminant hepatitis, liver cirrhosis, and other complications[1]. MSM are disproportionately affected by HCV infection[2], especially HIV-positive MSM with high-risk behaviors such as condomless anal sex or injection drug use [3]. A global systematic review found the prevalence of HCV in MSM living with HIV was 6.3% (95% CI: 5.3 to 7.5) compared to 1.5% (95% CI: 1.0 to 2.1) in HIV-negative MSM[4]. In China, a meta-analysis estimated that HCV prevalence was 1.2% and 8.4% among general MSM and MSM living with HIV, respectively[5].

Expanding HCV testing is critical for HCV control[3]. WHO recommends all MSM should receive HCV testing at least once every year[6]. Studies suggest that only 41% of Chinese MSM have been tested for HCV in their lifetime[7], which was 49% lower than the WHO’s target[8]. Previous studies showed that the low perception of HCV test and fear of stigma and discrimination from health care providers contributed to the low uptake of HCV testing among MSM[9]. Additionally, inconvenience and lack of privacy for facility-based HCV testing, unlinked systems for HIV and other STDs, and COVID-19 restrictions also led to low HCV testing uptake [10, 11]. In response to these gaps, the latest guideline from WHO strongly recommends HCVST as a potential method to enhance the uptake of HCV testing among MSM[12].

HCVST is a process in which an individual collects their specimen (blood or oral fluid), performs a simple rapid diagnostic test, and then interprets their result in a private setting, either alone or with someone they trust[12]. Several studies in Egypt[13] and Vietnam[14] have demonstrated high usability, acceptability, and feasibility of using HCVST among MSM, which provides a solid foundation to support the promotion of HCVST.

HCVST kits have been approved for HCV screening in China and are accessible through e-commerce platforms (Taobao and Jingdong). However, there are limited studies to evaluate the effectiveness and cost-effectiveness of HCVST compared to traditional facility-based testing. The purpose of this protocol is to design two parallel randomized controlled trials to evaluate the effectiveness and cost of providing HCVST on increasing the frequency of HCV testing compared with standard-of-care: one among HIV-negative MSM (trial 1) and one among MSM living with HIV (trial 2) in China.

**Methods**

**Study design**

Two parallel, unmasked, individual-level, randomized controlled trials will be conducted among MSM. All enrolled MSM will be offered to join one of the two trials according to their HIV status. In trial 1, we will enroll MSM who had an HIV-negative result based on their testing report in the past three months. In trial 2, we will enroll MSM who had an HIV-positive result determined by their testing report. In each trial, all participants will be randomly assigned in a 1:1 ratio into one of two arms: standard of care (control arm) and HCVST. The SOC arm will receive a community-based message and postcard to be encouraged to take anti-HCV IgG testing at local clinics. In addition to the message and postcard for men in the SOC arm, the HCVST arm will be provided with free HCVST kits during the study period. Participants of each arm will be followed for four weeks (Fig. 1). Preliminary data from a pilot study will be used to inform the final trial design.

**Study setting and population**

Participants are eligible if they are born biologically male, age 18 years or above, have anal sex with other men, report not testing for HCV in the past year, report at least one of the following risk factors in the past year (condomless anal sex or STD diagnosed or injection drug use), plan to stay in China for the next month and have a stable residence where they can securely receive a postal package. Participants are excluded if they are participating in similar clinical trials or other programs involving HCV testing or unwilling or unable to comply with all the requirements of the study. All enrolled participants will be divided into the two trials according to their HIV status. All participants will be recruited from seven MSM-led clinics run by MSM-focused CBOs in seven provinces located in different parts of China: East China (Shandong Province), South China (Guangdong Province), Northeast China (Liaoning Province), Northwest China (Qinghai Province), Central China (Hubei Province), Southwest China (Chongqing), and North China (Hebei Province). All the MSM-led clinics offer free HIV and syphilis testing and counseling for MSM. Staff at each CBO site also have rich experience in HIV/syphilis counseling and intervention. All the MSM living with HIV will be recruited offline, and HIV-negative MSM will be recruited by half of the online and offline ratios through CBOs. All study sites will follow the same research procedure.

All participants will be asked to finish a baseline survey for eligibility screening through Wenjuanxing (an online survey platform belonging to Changsha Ranxing Information Technology Co., Ltd, China). Online informed consent will be obtained before the baseline survey by clicking on a “button” indicating that the participant has read the consent form and agrees to participate in this research.

**Randomization and allocation**

All eligible individuals who complete the baseline survey and provide contact information will be assigned to one of the two arms in a 1:1 ratio using a randomized block design. A separate randomization procedure will be used for each CBO to generate 12 HCVST/SOC randomized assignments for trial 1 (HIV-negative MSM) and 12 HCVST/SOC randomized assignments for trial 2 (MSM living with HIV). The final randomization schedule will be generated by a statistician who is not involved in participant recruitment, using SAS version 9.4 (SAS Institute, Cary, NC, USA). Statisticians who analyze the data will be the only blinded people in this study.

**Arms and interventions**

Arms and interventions are summarized in Table 1.

**Table 1. Arms and interventions information**

| **Arm** | **Intervention** | **Intervention delivery** |
| --- | --- | --- |
| **Standard of care** | **Health promotion**:  1) community-engaged information about the risk of HCV infection (including a message and a postcard).  2) recommendation to undergo anti-HCV IgG testing at local clinics. | - Message and postcard will be sent at enrollment via WeChat. - Voluntary report of testing results to the platform. |
| **HCVST** | **Health promotion**:  1) community-engaged information about the risk of HCV infection (including a message and a postcard).  2) recommendation to undergo anti-HCV IgG testing at local clinics.  **Self-testing**: free home delivery of a self-testing package if participants apply for it. | - Message and postcard will be sent at enrollment via WeChat. - Provision of HCV self-testing service for free: self-testing package can be ordered online and sent to the participant through postal mail. Maximum one self-testing package per participant. - Voluntary report of testing results to the platform. |

***SOC arm***

Participants will receive a message and a postcard about basic HCV transmission and prevention knowledge through WeChat. The message uses concise language to encourage MSM to get tested for HCV. To better engage the MSM community and make the message more accessible, we invited three MSM-focused CBOs’ leaders and ten community members to create the message and finalize it based on their feedback. The postcard was developed through a national crowdsourcing contest in China in 2017 with vivid expressions to introduce knowledge about the risk of HCV transmission and infection. It was later proven to be effective in promoting HCV testing among MSM[15]. We will also provide recommendations for anti-HCV IgG testing at local clinics on the postcard.

***HCVST arm***

In this arm, participants will receive SOC arm interventions. Additionally, participants in this arm will be offered HCV self-testing kits for free. Participants can order self-testing packages online and apply for free home delivery through WeChat or Wenjuanxing and are allowed a maximum of one self-testing package for the duration of the study. Each HCV self-testing package contains equipment for blood sample collection, a quick HCV test, and a step-by-step pictorial instruction and operation video for using the self-testing kit. The provision of HCV self-testing service will last for four weeks. In this study, we will use the ABON HCV Hepatitis C Virus Antibody Rapid test kit, which has a sensitivity and specificity of 99.53% and 99.78%, respectively[16].

**Follow-up support**

An HCV counselor will be in charge of care support through WeChat or telephone (office hours: 8:00 am to 5:30 pm, Monday to Friday). Care support will include HCV pre-test counseling, instruction for the HCV self-testing kit, explanation of testing results, advice on testing results, and other relevant emergencies. All the inquiry logs will be kept. Participants are advised to inform the HCV counselor of any self-testing results and will be referred to the designated hospital for confirmatory laboratory testing and clinical examination based on the standard protocols of the respective clinics if necessary. If participants are diagnosed with HCV, further follow-up will include HCV confirmatory testing and treatment information.

**Outcome**

***Primary outcome***

The primary outcome is the HCV testing rate (including facility testing and self-testing) within four weeks, excluding confirmatory tests after a positive self-test. All testing results will use photo verification for validation. A participant will only be counted as having the primary outcome if the participant has submitted a photo, including age, test date, and anti-HCV IgG results, to the research team through WeChat or the online platform.

***Secondary outcomes***

We will additionally look at the incremental benefit of HCVST efficacy in promoting HCV testing, the number of newly identified cases of HCV, the linkage to HCV clinical care after self-testing, and HIV, chlamydia, and gonorrhea testing within four weeks.

We will examine cost-effectiveness alongside the trial. Using a decision-tree model, we will calculate the cost per person tested for HCV (including both self-testing and facility-based testing), cost per person self-tested for HCV, cost per person tested for HCV in a facility, and cost per person diagnosed with HCV. We will also examine the incremental cost-effectiveness ratio by comparing the two arms of the trials. We will conduct deterministic univariate sensitivity analysis and probabilistic sensitivity analysis to examine the robustness of our findings.

**Data collection**

***HCV testing record and results***

HCV testing results can be either from self-testing or facility-based testing. HCV self-testing will be confirmed by photo verification of the used testing kit. Facility-based HCV testing and the result will be confirmed by photo verification of the test report. Participants can securely upload testing results through WeChat or the Wenjuanxing Platform. A research assistant will conduct the verification process by checking the uploaded photos. Participants in all arms who upload photo verification will get 5 RMB (approximately equal to 0.8 USD dollars). All participants will be informed at enrolment that the cost of HCV testing, including facility-based HCV antibody testing and HCV self-testing during the four-week study period, can be reimbursed by providing a photo of the test report and receipt.

**Surveys**

***Baseline survey***

All participants will complete the baseline survey on the online survey platform (Wen Juan Xing, https://www.wjx.cn) at the time of enrollment. The baseline survey asks questions about their sociodemographic characteristics and sexual behaviors. After completing the survey, participants will receive 20 RMB (approximately 3 USD) as reimbursement.

***Follow-up survey***

All participants in the two arms will be asked to complete a brief online survey in four weeks. The follow-up survey will collect information about HCV testing frequency, time, location, method and result(s), and sexual behaviors within four weeks. Participants will receive 30 RMB (approximately 4 USD) for the follow-up survey.

***Cost data***

We will use a micro-costing approach to identify, measure and value costs associated with the trial arms. The perspective will be from the health provider, including the start-up costs (e.g., packaging, personnel training), consumables (HCV self-testing kits, standard of care testing supplies), capital costs (office equipment, building rental), and personnel costs. The time horizon will be the duration of the trial.

**Statistical methods**

All inferential tests will be two-sided and conducted at a significance level of *α*= 0.05 using SAS version 9.4 (SAS Institute, Cary, NC, USA). Baseline socio-demographic and behavioral characteristics will be presented separately by treatment arm and totaled across arms. Whenever feasible, data visualization will present individual-level data points as well as summary statistics.

***Sample size***

The sample size was calculated using Fisher’s exact test for two proportions using estimates from initial pilot study data (*n*=50 participants) in SAS version 9.4 (SAS Institute, Cary, NC, USA). Two of the 50 participants were not included in the estimates since they did not provide one-month follow-up data. In trial 1 (HIV-negative MSM), the HCV testing rates of the HCVST arm and the SOC arm were 78.9% and 10.5%, respectively. In trial 2 (MSM living with HIV), the HCV testing rate in the HCVST arm was 40.0%, and no participants in the SOC arm received testing for HCV.

For sample size calculations, we assumed equal sample sizes in HCVST and SOC (i.e., 1:1 allocation ratio), 90% power, the significance level of *α*= 0.05, two-sided testing, and 20% loss-to-follow-up (LTFU). The estimated required sample size for trial 1 (HIV-negative MSM) was *n*=17 in each arm (*n*=34 total). The estimated required sample size for trial 2 (MSM living with HIV) was *n*=27 in each arm (*n*=54 total).

Since the pilot study was relatively small (*n*=48 evaluable participants, 38 HIV-negative and 10 MSM living with HIV), the estimated effect sizes were possibly exaggerated. Based on our resources, we plan to enroll a sample size of 42 in each arm (*n*=84 total) for trial 1 (HIV-negative MSM) and a sample size of 42 in each arm (*n*=84 total) for trial 2 (MSM living with HIV). Thus, each CBO will enroll 24 participants: *n*=12 in trial 1 (HIV-negative MSM) and *n*=12 in trial 2 (MSM living with HIV). With this sample size and assuming a 10% testing rate in the SOC, we will be 90% powered to detect a difference of approximately 32% or higher in the HCV testing rate between the HCVST and SOC arms.

**Table 2. Sample size calculations**

|  | **HIV-negative MSM** | **MSM living with HIV** |
| --- | --- | --- |
| Proportion tested in HCVST | 0.79 (15/19) | 0.40 (2/5) |
| Proportion tested in SOC | 0.11 (2/19) | 0 (0/5) |
| Alpha | 0.05 | 0.05 |
| Power | 0.9 | 0.9 |
| Anticipated LTFU | 0.2 | 0.2 |
| Sample size in both arms  (With 20% LTFU) | 34 | 54 |

Abbreviations: Men who have sex with men (MSM), hepatitis C virus self-testing (HCVST), standard of care (SOC), loss-to-follow-up (LTFU).

***Analysis plan***

*Primary outcome*

The primary outcome of HCV testing rate (including facility testing and self-testing) within four weeks will be evaluated using a difference in proportions. We are interested in determining whether there is an overall difference in HCV testing rates between the arms. There may be differences in patient populations across provinces (CBOs), so we will adjust for CBOs in the analysis if there is evidence of such differences. The appropriate approach for such an analysis will depend on the frequency of testing in each arm. A model-based approach that uses identity link and binomial family can be used to adjust for CBOs if the frequencies are not too extreme (e.g., too few testers in SOC). Otherwise, a stratified test like Cochran–Mantel–Haenszel can be used to produce an adjusted *P*-value. We may also consider pooling data from CBOs that have low frequencies in the SOC arm (e.g., 0 or 1 tester) prior to adjusting for CBOs.

*Secondary outcomes*

Secondary outcomes of testing rates for HIV, chlamydia, and gonorrhea will be also be evaluated as a difference in proportions using the methods described for the primary outcome.

*Economic evaluation*

We will estimate the total unit and incremental unit cost for each arm in two trials. All costs will be categorized as startup costs, fixed costs, and variable costs in this study. Start-up costs refer to costs incurred in the process of study beginning, including the coordination of survey platforms. Fixed costs refer to costs that do not change with the number of participants throughout the trial period, including venue and equipment costs. Variable costs refer to costs that vary with the number of participants used and include HCVST kits, postage and other consumables. We will calculate all the costs for each arm, and then this cost will be divided by the number of MSM tested and the number of newly detected HCV cases in each study arm to calculate incremental unit costs. In addition, the univariate, multivariate, and probabilistic sensitivity analyses from a decision tree model will be used to examine uncertainty.

***Modified intention-to-treat and missing data***

The main analysis will be modified intention-to-treat in the sense that individuals will be included according to their randomization assignment, regardless of compliance; however, depending on the percent missingness, we will take two different approaches to handle missing data for those who did not complete the one-month follow-up survey. We anticipate the loss to follow-up will be less than 20% during four weeks. If the primary outcome is missing less than 11% of participants, then the main analysis will use a complete-case methodology. If the primary outcome is missing for 11% to 20% of participants, then we will utilize multiple imputation for our main analysis and present a sensitivity analysis using complete-case data.

**Ethical considerations**

The Institutional Review Board (IRB) approval was obtained from the Dermatology Hospital of Southern Medical University (2021046). All participants will be provided an online consent form before study initiation.

**Trial registration**

The study has been registered with the Chinese Clinical Trial Registry (trial ID ChiCTR2100048379).

**Discussion**

In this study, we will test two HCV testing models (SOC and HCVST) in two parallel randomized controlled trials among HIV-negative MSM and MSM living with HIV, respectively. According to WHO’s HCVST guidelines, HCVST has demonstrated good usability and feasibility in a series of observational studies[12]. Thus, we assume that HCVST can increase HCV testing uptake by addressing the structural barriers within traditional facility testing services. Our study is the first to utilize self-testing to examine HCV testing and linkage-to-care among HIV-negative MSM and MSM living with HIV. Findings from this study will contribute to the scarce data on the impact of HCVST on the promotion of routine HCV screening among MSM, which will help us better understand the safety, effectiveness, and acceptability of HCVST.

The major challenge will be verifying HCV testing results in this study. Some participants will undergo a facility-based HCV test or HCVST but may not provide feedback on their test results, which will lead to an underestimation of the rate of HCV testing. To increase the retrieval rate of validated testing proof, we will use WeChat to remind participants to take HCV testing and provide a cash reward of approximately 0.8 USD to participants who submit testing results or other test certification materials within four weeks. In addition, to make the message and postcard more accessible to MSM, we created the message based on three MSM-focused CBOs leaders and ten community members’ feedback. The postcard was developed through a national crowdsourcing contest in China in 2017 and has been effective in promoting HCV testing among MSM[15].

Several other challenges may be encountered during the study implementation. First, participants in the HCVST arm will be provided HCV self-testing kits for free, while participants in the SOC arm need to pay for their HCV test, which could lead to a different level of testing incentive and reduce the comparability between the two arms. Therefore, all the participants will be informed that the cost of any facility-based HCV testing in the four-week period can be reimbursed by providing their test report and receipt to the investigator. Second, HCVST may not adequately connect users with post-testing services, such as confirmatory testing and management of those who test users. In this study, all participants will be recruited through a local CBO, which could be helpful for the subsequent linkage to care. Third, the four weeks duration of follow-up will limit the ability of the study to assess the long-term effect of the intervention on HCV. Fourth, to protect the confidentiality of MSM, especially for MSM living with HIV, names and other specific identifying information will not be collected. All data will be encrypted and stored in remote repositories.

Another potential limitation of this study is the potential for sampling bias since the participants will be recruited through CBOs. Thus, this recruitment strategy will exclude those participants who are less likely to visit these CBOs[21]. Besides, all the recruitment sites are in the central district of cities, which may also lead to sampling bias and restrict the generalizability of results to MSM living in non-urban settings.

In conclusion, this study will compare the effectiveness and cost-effectiveness of HCVST and traditional facility testing services among HIV-negative and MSM living with HIV. Finding from this study will help explore whether HCVST can make up for the shortcomings of traditional facility testing services by improving the early diagnosis rate of HCV.

**List of abbreviations**

MSM: Men who have sex with men

HCV: Hepatitis C virus

HCVST: HCV self-testing

CBO: Community-based organizations

SOC: Standard of care

WHO: World Health Organization

**Declarations**

**Ethics approval and consent to participate**

This study was approved by the Ethical Committee of Dermatology Hospital of Southern Medical University. All participants will provide informed consent.

**Consent for publication**

Not applicable.

**Availability of data and materials**

The datasets analyzed during this study are not publicly available due to ethical and confidentiality reasons but are available from the corresponding author on reasonable request.

**Competing interests**

The authors declare that they have no competing interests.

**Funding**

This publication is supported by Medical Scientific Research Foundation of Guangdong Province, China (B2021297,B2022139).

**Authors’ contributions**

WC, JDT, ZPZ conceived the study. WC, PZZ, WQX and RXY led pilot and implementation optimization. AMW, JJO provided statistical expertise. WC, PZZ, WQX recruited participants. PZZ, JSW and SYM wrote initial draft of the paper. All authors read and authorized the final version.

**Acknowledgments**

Statistical support was provided by University of North Carolina at Chapel Hill Center for AIDS Research (CFAR), an NIH funded program P30 AI050410. We also thank Yen Chang and Katie R Mollan for providing statistical expertise.

|  |  |
| --- | --- |
|  | 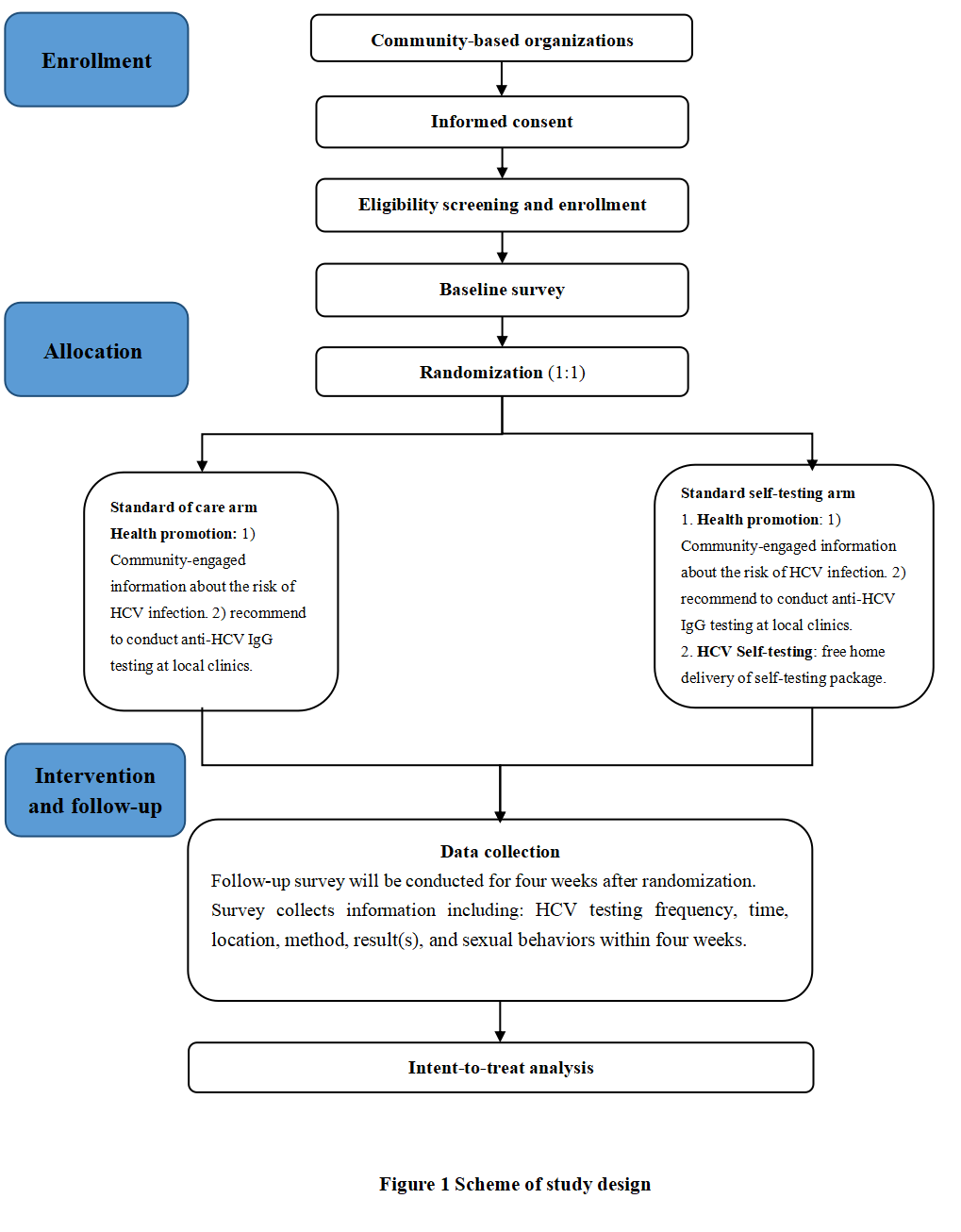 |

**Figure1 Scheme of study design**

**References:**

[1]. World Health Orgnization, Global Health Sector Strategies on HIV, Viral Hepatitis and Sexually Transmitted Infections 2022-2030.

[2]. Charre, C., et al., Hepatitis C virus spread from HIV-positive to HIV-negative men who have sex with men. PLOS ONE, 2018. 13(1): p. e0190340.

[3]. USCDC, Sexually Transmitted Infections Treatment Guidelines, 2021.

[4]. Jin, F., et al., Prevalence and incidence of hepatitis C virus infection in men who have sex with men: a systematic review and meta-analysis. The lancet. Gastroenterology & hepatology, 2021. 6(1): p. 39.

[5]. Chow, E.P.F., et al., Disparities and risks of sexually transmissible infections among men who have sex with men in China: a meta-analysis and data synthesis. PloS one, 2014. 9(2): p. e89959-e89959.

[6]. Pan, S.W., et al., Systematic review of innovation design contests for health: spurring innovation and mass engagement. BMJ Innov, 2017. 3: p. 227-237.

[7]. Fitzpatrick, T., et al., HBV and HCV test uptake and correlates among men who have sex with men in China: a nationwide cross-sectional online survey. Sexually Transmitted Infections, 2018. 94(7): p. 502-507.

[8]. Li, M., H. Zhuang and L. Wei, How would China achieve WHO's target of eliminating HCV by 2030? Expert Rev Anti Infect Ther, 2019. 17(10): p. 763-773.

[9]. Wang, R., et al., Barriers to uptake of hepatitis C virus (HCV) health intervention among men who have sex with men in Southwest China: A qualitative study. Health Soc Care Community, 2021. 29(2): p. 445-452.

[10]. Li, K.T., et al., Pay-it-forward strategy to enhance uptake of dual gonorrhea and chlamydia testing among men who have sex with men in China: a pragmatic, quasi-experimental study. The Lancet infectious diseases, 2019. 19(1): p. 76-82.

[11]. Wang, C., et al., Syphilis Self-testing: A Nationwide Pragmatic Study Among Men Who Have Sex With Men in China. Clinical Infectious Diseases, 2020. 70(10): p. 2178-2186.

[12]. World Health Orgnization, Recommendations and guidance on hepatitis C virus self-testing,2021.

[13]. Reipold, E.I., et al., Usability and acceptability of self-testing for hepatitis C virus infection among the general population in the Nile Delta region of Egypt. BMC Public Health, 2021. 21(1): p. 1188.

[14]. Nguyen, L.T., et al., Acceptability and Usability of HCV Self-Testing in High Risk Populations in Vietnam. Diagnostics, 2021. 11(2): p. 377.

[15]. Fitzpatrick, T., et al., A crowdsourced intervention to promote hepatitis B and C testing among men who have sex with men in China: A nationwide online randomized controlled trial. EClinicalMedicine, 2019. 16: p. 64-73.

[16]. Abon Biopharm.Hepatitis C Virus Rapid Test Device (Whole Blood/Serum/Plasma) Package Insert.https://www.globalpointofcare.abbott/en/product-details/abon.html.

[17]. Cheng, W., et al., Promoting routine syphilis screening among men who have sex with men in China: study protocol for a randomised controlled trial of syphilis self-testing and lottery incentive. BMC Infectious Diseases, 2020. 20(1).

[18]. Wang, R., et al., Barriers to uptake of hepatitis C virus (HCV) health intervention among men who have sex with men in Southwest China: A qualitative study. Health & Social Care in the Community, 2021. 29(2): p. 445-452.

[19]. Wang, Z., et al., Factors predicting first-time hepatitis C virus testing uptake among men who have sex with men in China: an observational prospective cohort study. Sex Transm Infect, 2020. 96(4): p. 258-264.

[20]. Tang, W., et al., Crowdsourcing to expand HIV testing among men who have sex with men in China: A closed cohort stepped wedge cluster randomized controlled trial. PLOS Medicine, 2018. 15(8): p. e1002645.

[21]. Wang, Y., et al., Social network distribution of syphilis self-testing among men who have sex with men in China: study protocol for a cluster randomized control trial. BMC Infect Dis, 2021. 21(1): p. 491.
