# Supplementary material for: Expanding hepatitis C virus test uptake using self-testing among men who have sex with men in China: two parallel randomized controlled trials
Source: BMC Med. 2023 Jul 28;21:279. doi: 10.1186/s12916-023-02981-w (PMC10386771; doi:10.1186/s12916-023-02981-w)

Additional file 3

# Figure S1. Manufacturer-supplied step-by-step instructions


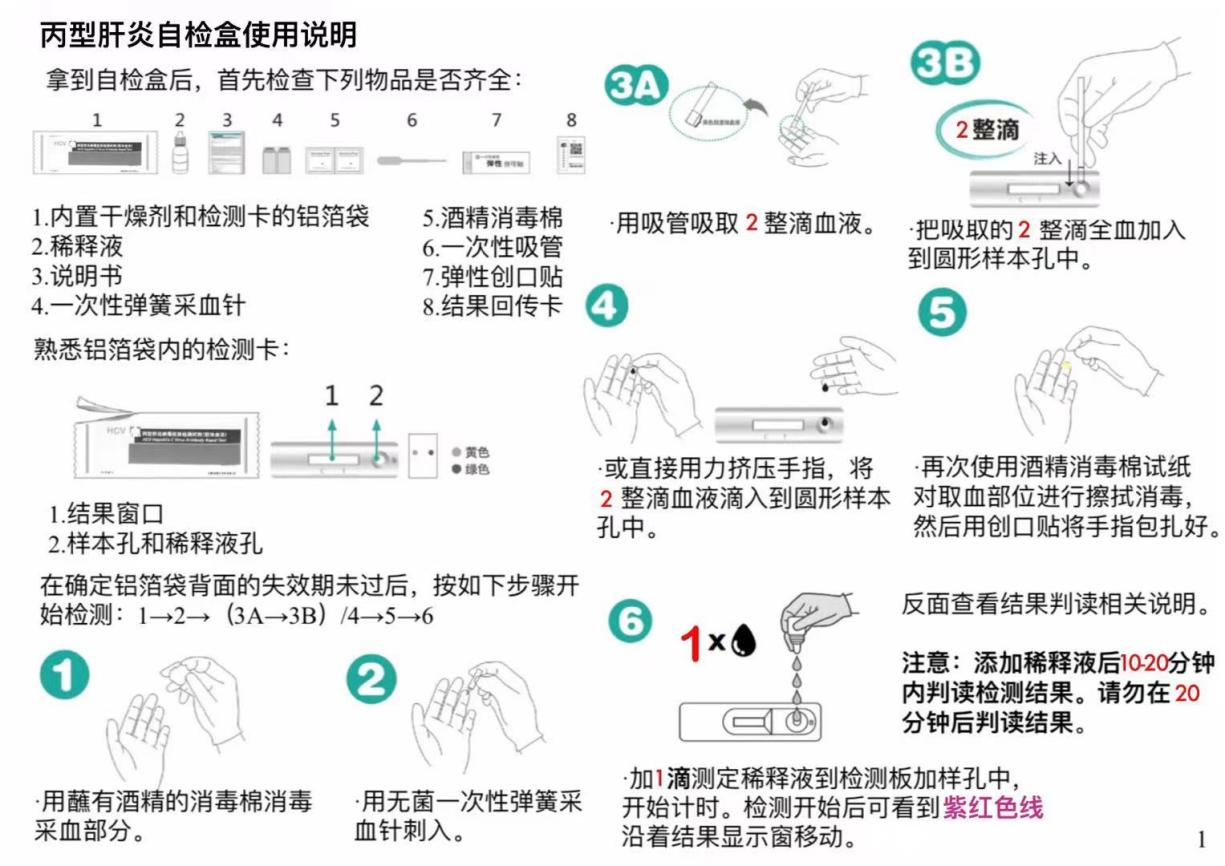


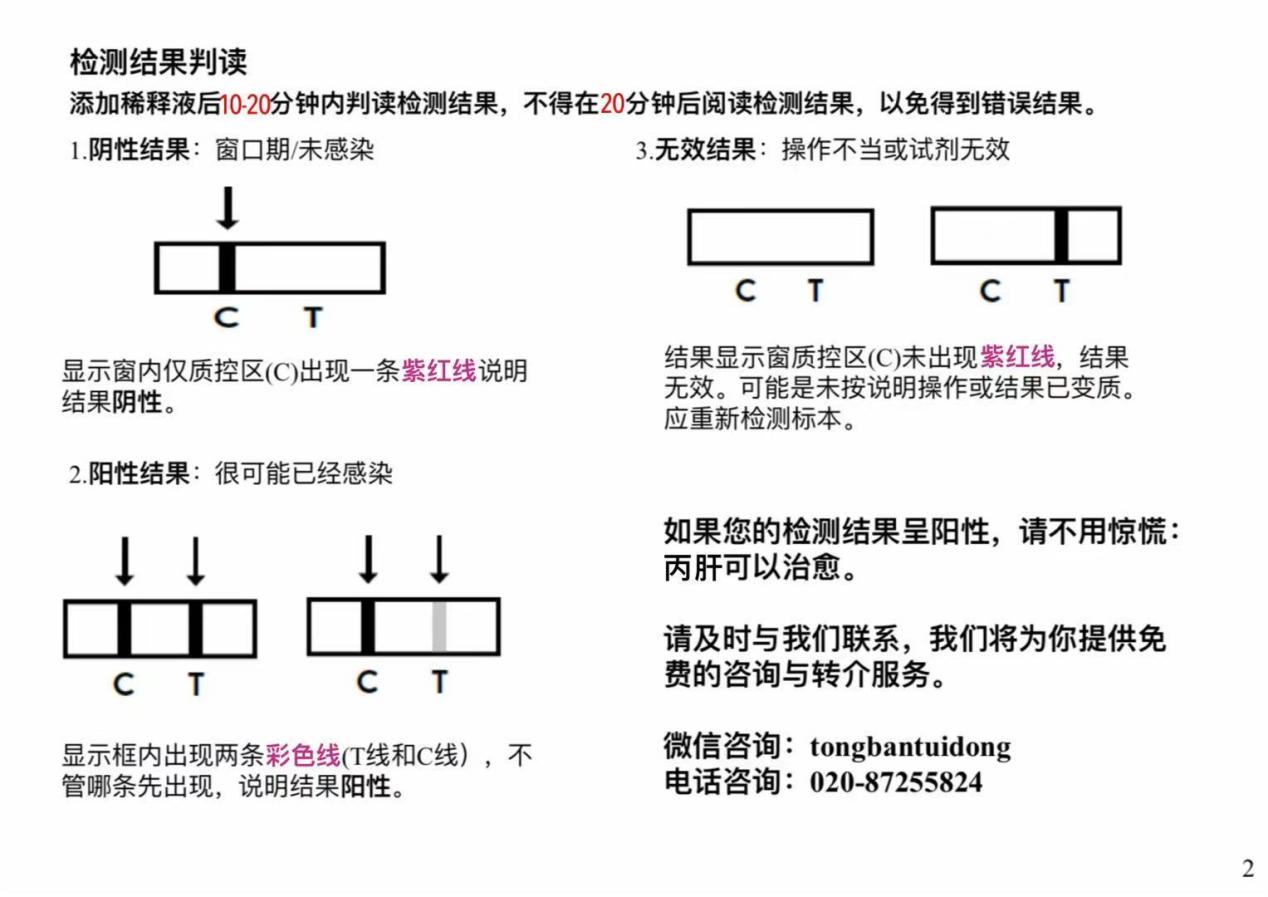


# Figure S2. Result report card

For the standard HCV self-testing arm


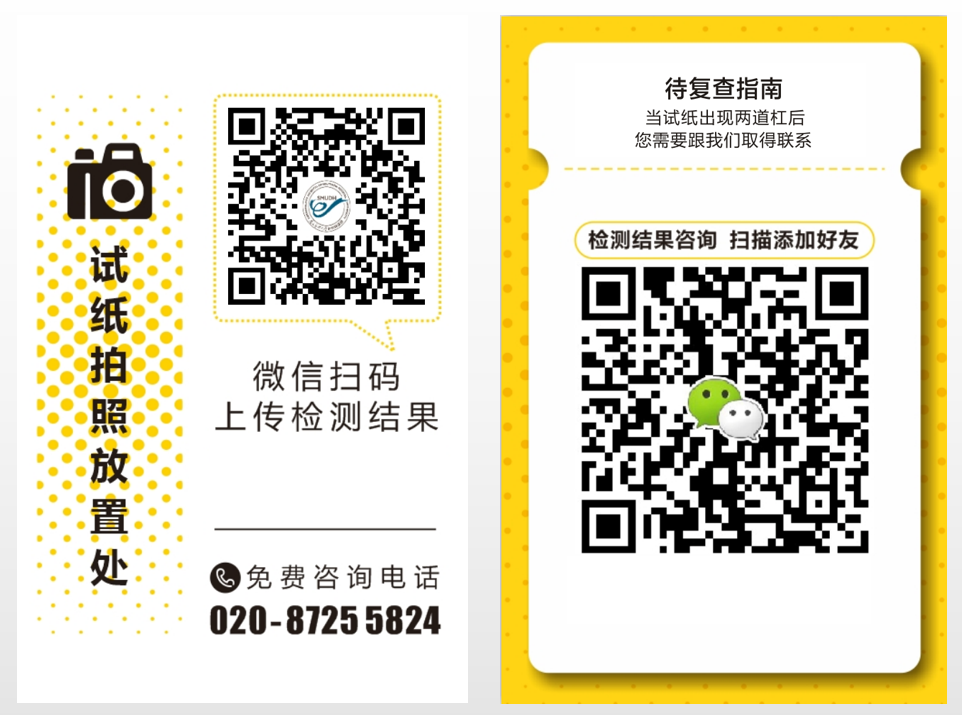

Supplement: Supplementary file 3 — Additional file 3: Fig. S1. Manufacturer-supplied step-by-step instructions. Fig. S2. Result report card. [file 12916_2023_2981_MOESM3_ESM.docx]
