# Supplementary material for: Expanding hepatitis C virus test uptake using self-testing among men who have sex with men in China: two parallel randomized controlled trials
Source: BMC Med. 2023 Jul 28;21:279. doi: 10.1186/s12916-023-02981-w (PMC10386771; doi:10.1186/s12916-023-02981-w)
Supplement: Supplementary file 4 — Additional file 4: Table S1. Baseline characteristics of study participants stratified by loss-to-follow-up in the HCV Self-Testing Randomized Controlled Trial in China in 2020. Table S2. Adverse outcome related to HCV self-testing characteristics among MSM in China (n=2). Table S3. Costs items (in 2022 USD) over a 2-month time-horizon. [file 12916_2023_2981_MOESM4_ESM.docx]

**Additional file** **4**

# Table S1. Baseline characteristics of study participants stratified by loss-to-follow-up in the HCV self-testing Randomized Controlled Trial in China in 2020.

|  | **Men who were HIV negative**  **(trial 1)** | | | **Men who were HIV positive**  **(trial 2)** | | |
| --- | --- | --- | --- | --- | --- | --- |
|  | **Lost-to-follow-up (n=10)^a^** | **Completed follow-up survey (n=74)^a^** | **p-value^b^** | **Lost-to-follow-up**  **(n=9)^a^** | **Completed follow-up survey (n=75)^a^** | **p-value^b^** |
| **Age (years)** |  |  | 0·440 |  |  | 0·480 |
| ≤30 | 7/10 (70·0) | 59/74 (79·7) |  | 4/9 (44·4) | 45/75 (60·0) |  |
| >30 | 3/10 (30·0) | 15/74 (20·3) |  | 5/9 (55·6) | 30/75 (40·0) |  |
| Mean (SD) | 29·5 (10·8) | 26·6 (6·9) |  | 33·0 (7·9) | 29·7 (7·6) |  |
| **Marital status** |  |  | **1·000** |  |  | **0·015** |
| Never married | 9/10 (90·0) | 65/74 (87·8) |  | 5/9 (55·6) | 68/75 (90·7) |  |
| Ever married | 1/10 (10·0) | 9/74 (12·2) |  | 4/9 (44·4) | 7/75 (9·3) |  |
| **Annual income (US$)** |  |  | **0·020** |  |  | 0·736 |
| <2800 | 2/10 (20·0) | 12/74 (16·2) |  | 2/9 (22·2) | 11/75 (14·7) |  |
| 2800-5600 | 0/10 (0·0) | 8/74 (10·8) |  | 2/9 (22·2) | 10/75 (13·3) |  |
| 5601-9500 | 1/10 (10·0) | 35/74 (47·3) |  | 2/9 (22·2) | 27/75 (36·0) |  |
| 9501-15000 | 4/10 (40·0) | 13/74 (17·6) |  | 1/9 (11·1) | 16/75 (21·3) |  |
| ≥15001 | 3/10 (30·0) | 6/74 (8·1) |  | 2/9 (22·2) | 11/75 (14·7) |  |
| **Highest education** |  |  | 0·511 |  |  | 0·715 |
| High school or below | 7/10 (70·0) | 42/74 (56·8) |  | 5/9 (55·6) | 49/75 (65·3) |  |
| College or beyond | 3/10 (30·0) | 32/74 (43·2) |  | 4/9 (44·4) | 26/75 (34·7) |  |
| **Sexual orientation** |  |  | 0·450 |  |  | 0·624 |
| Gay/homosexual | 6/10 (60·0) | 55/74 (74·3) |  | 7/9 (77·8) | 64/75 (85·3) |  |
| Bisexual or other | 4/10 (40·0) | 19/74 (25·7) |  | 2/9 (22·2) | 11/75 (14·7) |  |
| **Disclosure as MSM to family, friends, or health-care professionals** | | | 0·303 |  |  | 1·000 |
| Never | 5/10 (50·0) | 24/74 (32·4) |  | 2/9 (22·2) | 20/75 (26·7) |  |
| Ever | 5/10 (50·0) | 50/74 (67·6) |  | 7/9 (77·8) | 55/75 (73·3) |  |
| **Anal sex without use of condom in the past 6 months** | | | 1·000 |  |  | 0·169 |
| No | 1/4 (25·0) | 12/62 (19·4) |  | 4/6 (66·7) | 19/62 (30·6) |  |
| Yes | 3/4 (75·0) | 50/62 (80·6) |  | 2/6 (33·3) | 43/62 (69·4) |  |
| **Number of male sex partners in the past three months** | | | 0·501 |  |  | 0·080 |
| 0-1 | 3/10 (30·0) | 34/74 (45·9) |  | 5/9 (55·6) | 27/75 (36·0) |  |
| Multiple | 7/10 (70·0) | 40/74 (54·1) |  | 4/9 (44·4) | 48/75 (64·0) |  |
| **Ever tested for HBV** |  |  | 0·098 |  |  | 0·292 |
| No | 3/10 (30·0) | 44/74 (59·5) |  | 3/9 (33·3) | 42/75 (56·0) |  |
| Yes | 7/10 (70·0) | 30/74 (40·5) |  | 6/9 (66·7) | 33/75 (44·0) |  |

^a^Data are n/N (%) unless otherwise indicated. ^b^p-values computed using a two-sided Fisher’s exact test.

# Table S2. Adverse outcome related to HCV self-testing characteristics among MSM in China (n=2).

| **Item** | **n/N (%)** |
| --- | --- |
| **Have you ever been pressured/forced to self-test for HCV?** | 2/58 (3·45) |
| **Location where HCV self-testing coercion happened** |  |
| Your own home | 1/2 (50·0) |
| Health-care Facility | 1/2 (50·0) |
| **Relationship with the person who coerced you** |  |
| Fixed male sexual partner | 1/2 (50·0) |
| Other | 1/2 (50·0) |
| **Types of coercion before HCV self-testing** |  |
| Actual physical violence | 1/2 (50·0) |
| Psychological abuse | 1/2 (50·0) |
| **Types of coercion after getting self-testing results** |  |
| Physical violence | 1/2 (50·0) |
| Other | 1/2 (50·0) |

# Table S3. Costs items (in 2022 USD) over a 2-month time-horizon

| **Study arm** | **Cost item** | **Unit cost ($)** | **Source** |
| --- | --- | --- | --- |
| ***Standard-of-care*** |  |  |  |
| - Fixed costs | *Recruitment of men from NGO* | 659 | Trial invoices |
|  | *Personnel costs for staff* | 471 | Trial invoices |
|  | *Internet use* | 31 | Personal communication with local staff |
|  | *Utilities (electricity)* | 8 | Personal communication with local staff |
|  | *Property management* | 13 | Personal communication with local staff |
|  | *Telephone bill* | 8 | Personal communication with local staff |
|  | *Rental of WenJuanXing platform for data* | 38 | Personal communication with local staff |
|  | *Building rental* | 63 | Personal communication with local staff |
|  | *Computer^1^* | 4 | Trial invoices |
|  | *Desk^2^* | 0.72 | Trial invoices |
|  | *Chairs^2^* | 0.39 | Trial invoices |
| - Recurrent costs | ***Control group*** |  |  |
|  | *HCV test* | 4 | Dermatology Hospital of Southern Medical University |
|  | *Patient registration at clinic* | 2 | Dermatology Hospital of Southern Medical University |
|  | *Doctor’s time to see patient for HCV test (5 minutes)* | 1 | Average hourly wage of doctors $11 |
|  | ***HCVST arm*** |  |  |
|  | *Boxes* | 0.44 | Trial invoices |
|  | *Instruction book* | 0.16 | Trial invoices |
|  | *HCV self-test kit* | 1.57 | Trial invoices |
|  | *Blood taking needle* | 0.07 | Trial invoices |
|  | *Band-aid* | 0.01 | Trial invoices |
|  | *Alcohol swab* | 0.01 | Trial invoices |
|  | *Result upload card* | 0.06 | Trial invoices |
|  | *Listing page* | 0.03 | Trial invoices |
|  | *Delivery cost of self-test kit* | 2.16 | Trial invoices |

^1^ *The cost of a computer was annualised over a seven-year period at a discount rate of 3%, and the 2-month pro-rata costs valued here.*

^2^ *The cost of desk and chairs was annualised over a five-year period at a discount rate of 3% and the 2-month pro-rata costs valued here.*
